# ABC and VED Analysis of the Pharmacy Store of a Tertiary Care Teaching, Research and Referral Healthcare Institute of India

**DOI:** 10.4103/0975-1483.63170

**Published:** 2010

**Authors:** M Devnani, AK Gupta, R Nigah

**Affiliations:** *Department of Hospital Administration, Post Graduate Institute of Medical Education and Research (PGIMER), Chandigarh, India*; 1 *Department of Pharmacy, Post Graduate Institute of Medical Education and Research (PGIMER), Chandigarh, India*

**Keywords:** ABC analysis, ABC-VED matrix, inventory management, pharmacy, VED analysis

## Abstract

The ABC and VED (vital, essential, desirable) analysis of the pharmacy store of Post Graduate Institute of Medical Education and Research (PGIMER), Chandigarh, India, was conducted to identify the categories of items needing stringent management control. The annual consumption and expenditure incurred on each item of pharmacy for the year 2007-08 was analyzed and inventory control techniques, i.e. ABC, VED and ABC-VED matrix analysis, were applied. The drug formulary of the pharmacy consisted of 421 items. The total annual drug expenditure (ADE) on items issued in 2007-08 was Rs. 40,012,612. ABC analysis revealed 13.78%, 21.85% and 64.37% items as A, B and C category items, respectively, accounting for 69.97%, 19.95% and 10.08% of ADE of the pharmacy. VED analysis showed 12.11%, 59.38% and 28.51% items as V, E, and D category items, respectively, accounting for 17.14%, 72.38% and 10.48% of ADE of the pharmacy. On ABC-VED matrix analysis, 22.09%, 54.63% and 23.28% items were found to be category I, II and III items, respectively, accounting for 74.21%, 22.23% and 3.56% of ADE of the pharmacy. The ABC and VED techniques need to be adopted as a routine practice for optimal use of resources and elimination of out-of-stock situations in the hospital pharmacy.

## INTRODUCTION

About one-third of the annual hospital budget is spent on buying materials and supplies, including medicines.[1] The pharmacy is one of the most extensively used therapeutic facilities of the hospital and one of the few areas where a large amount of money is spent on purchases on a recurring basis. This emphasizes the need for planning, designing and organizing the pharmacy in a manner that results in efficient clinical and administrative services.[2] The goal of the hospital supply system is to ensure that there is adequate stock of the required items so that an uninterrupted supply of all essential items is maintained. A study conducted by the Department of Personnel and Administrative Reforms in India has revealed that not only does the quantity of medicines received fall short of the requirement but also the supply is often erratic. Even common medicines are out of stock and remain so for a considerable period.[3] Of the various explanations for non-availability of even simple medicines in the third world countries, a large number are related to materials management. A study from a 1,500-bedded state-funded hospital has claimed that review and control measures for expensive drugs brought about 20% savings.[4]

Inventory control in hospital pharmacy is very essential in a developing country like India.[5] As resources are limited, it is essential that the existing resources be appropriately utilized. With the existing drug budget, if rational drug use and improved drug management practices are followed, more number of patients can be served. It is essential that health managers use scientific methods to maximize their returns from investment at a minimal cost.[5–8]

Drug inventory management stresses on cost containment and improved efficiency.[9] Each item may be considered critical and there is a perceived need to supply very high levels of service.[10] There is no denying that stocking hospital pharmaceuticals and supplies can be expensive and tie up a lot of capital, and bringing efficiencies to such important cost drivers - often 30-40% of a hospital’s budget - can present meaningful savings.[11] Thus, a hospital materials manager must establish efficient inventory system policies for normal operating conditions that also ensure the hospital’s ability to meet emergency demand conditions.[12] But, it is impossible and unnecessary too to monitor every drug used in the health system. High-cost and high-volume drugs come in priority, whose intervention is likely to cause the greatest clinical and economic impact. In the whole process, it is important to trace the costliest medicinal products first, those that consume the major portion of the budget, and then design a strategy to further study and identify their use pattern. The study of use pattern will help in designing appropriate corrective measures. ABC analysis is an important tool used worldwide, identifying items that need greater attention for control.[5–8,13]

ABC analysis is a method of classifying items or activities according to their relative importance. It is also known as “separating the vital few from the trivial many” because, for any group of things that contribute to a common effect, a relatively few contributors account for a majority of the effects. The analysis classifies the items into three categories: the first 10-15% of the items account for approximately 70% of cumulative value (cost) (category A), 20-25% are category B items that account for a further 20% of the cumulative value and the remaining 65-70% are category C items, amounting for a mere 10% of the total value.[5–9,14–16]

The limitation of ABC analysis is that it is based only on monetary value and the rate of consumption of the item. In a hospital, an item of low monetary value and consumption may be very vital or even life saving. Their importance cannot be overlooked simply because they do not appear in category A. Therefore, another parameter of the materials is their criticality.[7]

VED analysis is based on critical values and shortage cost of the item. Based on their criticality, the items could be classified into three categories: vital, essential and desirable. There could be serious functional dislocation of patient care services in hospital when vital drugs are not available even for a short period. If essential items are not available beyond a few days or a week, the functioning of the hospital can be adversely affected. The shortage of desirable items would not adversely affect patient care or hospital functioning even if shortage is prolonged.[5,7,17]

A combination of ABC and VED analysis (ABC-VED matrix) can be gainfully employed to evolve a meaningful control over the material supplies. Category I includes all vital and expensive items (AV, BV, CV, AE, AD). Category II includes the remaining items of the E and B groups (BE, CE, BD). Category III includes the desirable and cheaper group of items (CD).[17]

In the present study, ABC, VED and ABC-VED matrix analysis of the pharmacy store of PGIMER, Chandigarh (a 1,500 bed tertiary care teaching, research and referral health institute catering to the major portion of northern India), was performed to identify the categories of drugs needing stringent management control.

The specific objectives of this study were to: (1) analyze the annual consumption of items of pharmacy and expenditure incurred on them for the year 2007-08, (2) evolve a priority system based on ABC and VED and ABC-VED matrix analysis, (3) identify the item categories requiring greater supervisory monitoring.

## MATERIALS AND METHODS

The data of annual consumption and expenditure incurred on each item of the pharmacy for the financial year 2007-08 were collected. The data were then transcribed in an MS Excel spreadsheet. The statistical analysis was carried out using the MS Excel statistical functions.

### ABC analysis

The annual expenditure of individual items was arranged in descending order. The cumulative cost of all the items was calculated. The cumulative percentage of expenditure and the cumulative percentage of number of items were calculated. This list was then subdivided into three categories: A, B and C, based on the cumulative cost percentage of 70%, 20% and 10%, respectively.

### VED analysis

The VED criticality analysis of all the listed items was performed by classifying the items into vital (V), essential (E) and desirable (D) categories. The items critically needed for the survival of the patients and those that must be available at all times were included in the V category. The items with a lower criticality need and those that may be available in the hospital were included in the E group. The remaining items with lowest criticality, the shortage of which would not be detrimental to the health of the patients, were included in the D group. The VED status of each item was discussed with justification by a group comprising of physician, surgeon, pediatrician and pharmacist.

### ABC-VED matrix analysis

The ABC-VED matrix was formulated by cross-tabulating the ABC and VED analysis. From the resultant combination, three categories were classified (I, II and III). Category I was constituted by items belonging to AV, AE, AD, BV and CV subcategories. The BE, CE and BD subcategories constituted category II, and the remaining items in the CD subcategory constituted category III. In these subcategories, the first alphabet denotes its place in the ABC analysis, while the second alphabet stands for its place in the VED analysis.

## RESULTS

The drug formulary of the hospital consisted of 421 items. The total ADE of the pharmacy on items issued in 2007-08 was Rs. 40,012,612.

### ABC analysis

**Figure 1:**
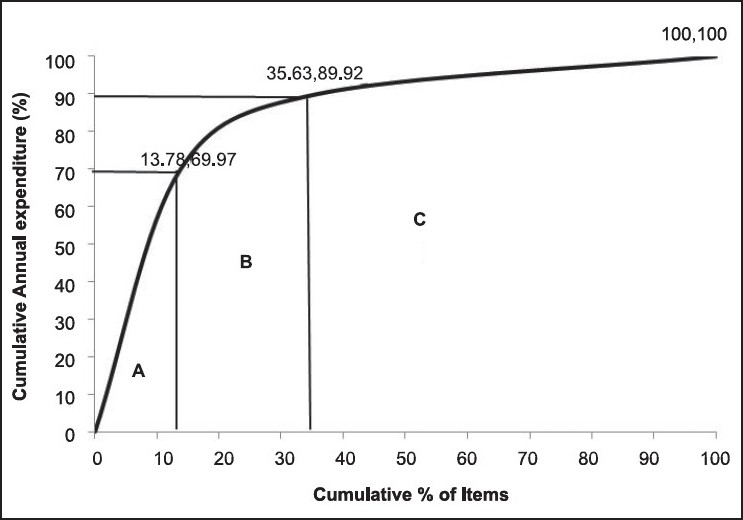
ABC analysis cumulative curve (2007-08)

On ABC analysis, 13.78% (58), 21.85% (92) and 64.37% (271) items were found to be A, B and C category items, respectively, amounting for 69.97% (Rs. 27,996,865), 19.95% (Rs. 7,981,331) and 10.08% (Rs. 4,034,416) of ADE of the pharmacy [Table 1 and Figure 1]. The cut-offs were not exactly at 70/20/10%, and differed marginally, which is permissible.[18]

**Table 1:** The ABC, VED and ABC-VED matrix analysis of the PGIMER pharmacy (2007-08)

Category	No. of items	% of items	ADE (Rs.)	% of ADE of the pharmacy
A	58	13.78	27,996,865	69.97
B	92	21.85	7,981,331	19.95
C	271	64.37	4,034,416	10.08
V	51	12.11	6,857,814	17.14
E	250	59.38	28,963,447	72.38
D	120	28.51	4,191,351	10.48
I	93	22.09	29,691,956	74.21
II	230	54.63	8,895,160	22.23
III	98	23.28	1,425,496	3.56

### VED analysis

**Figure 2:**
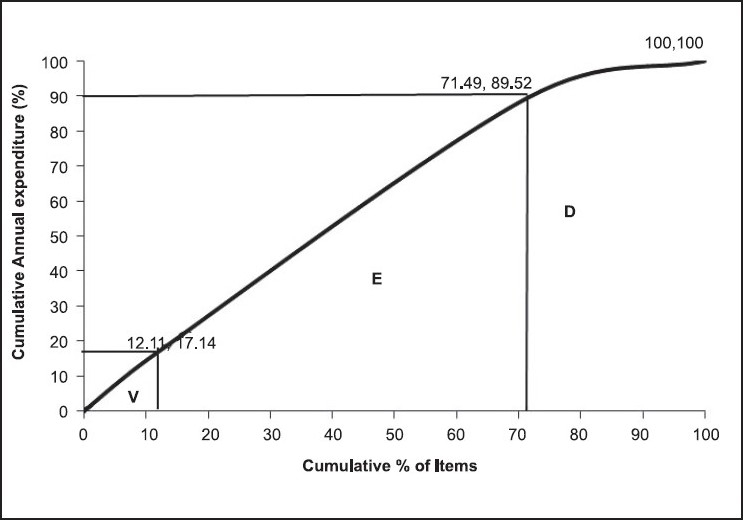
VED analysis cumulative curve (2007-08)

The findings of the VED analysis of the present study are shown in Table 1 and Figure 2. About 12.11% (51), 59.38% (250) and 28.51% (120) items were found to be V, E and D category items, respectively, amounting for 17.14% (Rs. 6,857,814), 72.38% (Rs. 28,963,447) and 10.48% (Rs. 4,191,351) of ADE of the pharmacy.

### ABC-VED matrix analysis

Table 2 shows the ABC-VED matrix analysis. Nine different subcategories (AV, AE, AD, BV, BE, BD, CV, CE and CD) were studied using this analysis. These nine were further grouped into three main categories, categories I, II and III [Table 1].

**Table 2:** ABC-VED matrix analysis of the PGIMER pharmacy (2007-08)

	V				E				D			
	No.	%	Annual expenditure (Rs.)	%	No.	%	Annual expenditure (Rs.)	%	No.	%	Annual expenditure (Rs.)	%
A	16	3.80	5,162,722	12.90	36	8.55	21,495,547	53.72	6	1.43	1,338,595	3.34
B	16	3.80	1,398,518	3.50	60	14.25	5,155,508	12.88	16	3.80	1,427,260	3.57
C	19	4.51	296,574	0.74	154	36.58	2,312,392	5.78	98	23.28	1,425,495	3.57
Total	51	12.11	6,857,814	17.14	250	59.38	28,963,447	72.38	120	28.51	4,191,351	10.48

Note: % indicates percentage of total items in drug list/total ADE of the pharmacy.

**Figure 3:**
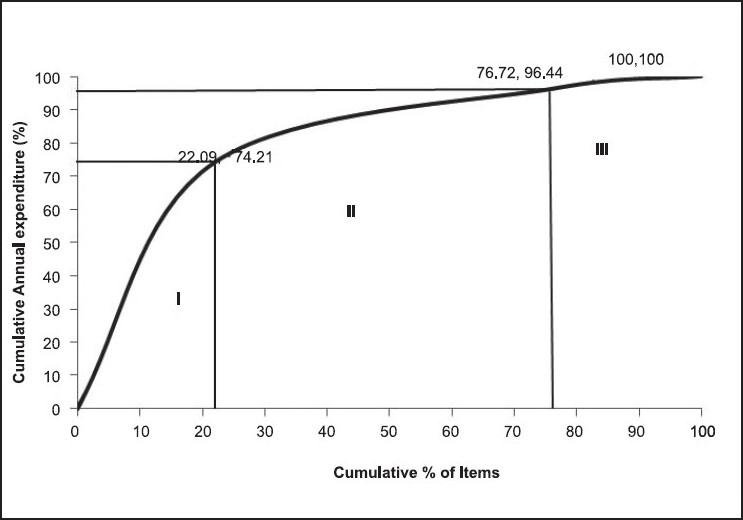
ABC-VED matrix cumulative curve (2007-08)

There were 93 (22.09%) items in category I, 230 (54.63%) items in category II and 98 (23.28%) items in category III, amounting for 74.21% (Rs. 29,691,956), 22.23% (Rs. 8,895,160) and 3.56% (Rs. 1,425,496) of ADE of the pharmacy, respectively [Table 1 and Figure 3].

## DISCUSSION

Provision of care in tertiary care hospitals is sensitive to the timely availability of facilities, including drugs. In case of drugs, besides the criticality factor, the cost factor must also be taken into consideration, as can be seen from our study, where about 10% of the drugs consumed about 70% of ADE of the pharmacy. This is the group requiring greater monitoring as it has fewer drugs consuming most of the money. We also noted that not all the drugs in this group were vital or essential. It also had drugs from the desirable category. Categorization of drugs by the ABC-VED matrix model helps to narrow down on fewer drugs requiring stringent control.

### ABC analysis

The present study showed that if ABC analysis is considered alone for drug inventory, it would help effectively control the recommended 58 (13.78%) items in the A category, with almost 70% of ADE of the pharmacy, but it would compromise on the availability of items of vital nature from B and C categories (35 items, 8.31%). The results of the study are comparable with similar studies conducted in India [Table 3].[9,17,19–20]

**Table 3:** Comparison of ABC, VED and ABC-VED matrix analysis of different studies in India

Category	Present study	GMCH, Goa study[17]	Service hosp., AFI study[20]	GMCH, Nagpur study[9]	CGHS study[19]
A	13.78	12.93	14.46	10.76	17.81
B	21.85	19.54	22.46	20.63	22.60
C	64.37	67.53	63.08	68.61	59.59
V	12.11	12.36	7.39	23.76	5.14
E	59.38	47.12	49.23	38.12	58.90
D	28.51	40.52	43.38	38.12	35.96
I	22.09	22.99	20.92	29.15	21.58
II	54.63	41.67	48.92	41.26	56.16
III	23.28	35.34	30.16	29.59	22.26

Note: All figures are in %, GMCH, Government Medical College and Hospital; AFI, Armed Forces of India; CGHS, Central Government Health Services of India.

### VED analysis

If VED analysis alone is considered, ideal control can be exercised on the identified vital and/or essential items, accounting for 89.52% of ADE of the pharmacy. However, category A also contains six desirable items with 3.34% of ADE of the pharmacy and hence it is not possible to ignore the desirable group completely. The comparison with similar studies in India showed high variation in the percentage of vital, essential and desirable items [Table 3].[9,17,19–20] This could be because different institutes have different service profiles, depending on the specialty services available.

### ABC-VED matrix analysis

In a combination of ABC and VED analysis, the resultant matrix makes it possible to focus on 93 (22.09%) items belonging to category I for strict managerial control as these items are either expensive or vital. The annual expenditure of these items was 74.21% of ADE of the pharmacy. AV, AE and BV subgroups of category I consist of 68 items (16.15%) that are expensive (70.12% of ADE), and their being out of stock is unacceptable as they are either vital or essential. To prevent locking up of capital due to these items, low buffer stock needs to be maintained while keeping a strict vigil on the consumption level and the stock in hand. A two-bin method of ordering needs to be followed for these as this will eliminate the risk of item shortage. CV items (19, 4.51%) are drugs of low cost but high criticality and take up 0.74% of ADE of the pharmacy. Because this amount is negligible, these items can be procured once a year and stocked as their carrying cost is low.

AD items (only six, 1.43%) consume 3.34% of the ADE. These items should be monitored for economic order quality, and their order placement must be made after careful study of the need. Rational use of items in this subgroup, including their removal from the list if possible, can bring about substantial savings without affecting patient care.

Category II items (230, 54.63%) consumes 22.23% of the ADE. These items can be ordered once or twice a year, thereby saving on ordering cost and reducing management hassles at a moderate carrying cost and without blocking substantial capital. Category III items (98, 23.28%) consume 3.57% of the ADE. These items can also be ordered once or twice a year, thereby saving on ordering cost at a moderate carrying cost and without blocking substantial capital. The comparison with similar studies in India is shown in Table 3.[9,17,19–20]

## CONCLUSION

During the year 2007-08, items of approximately Rs. 40,012,612 were issued by the pharmacy store of PGIMER. This necessitates application of scientific inventory management tools for effective and efficient management of the pharmacy stores, efficient priority setting, decision making in purchase and distribution of specific items and close supervision on items belonging to important categories. ABC and VED analysis identifies the drugs requiring stringent control for optimal use of funds and elimination of out-of-stock situations in the pharmacy.
